# An Attempt to Explain the Principal Means Which the System Adopts in Its Efforts to Relieve Itself, When Labouring under Affections Attended with Increased Action of the Circulating Fluids; More Particularly as They Are Connected with the Existence of the Buffy Coat in the Blood

**Published:** 1829-11

**Authors:** Andrew Blake

**Affiliations:** Member of the Royal College of Surgeons, and Surgeon to his Majesty's 7th Regiment of Dragoon Guards.


### BUFFY COAT IN THE BLOOD.

*An Attempt to explain the principal Means which the System adopts in its Efforts to relieve itself, when labouring under Affections attended with increased Action of the circulating Fluids; more particularly as they are connected with the existence of the Buffy Coat in the Blood.* By ANDREW BLAKE, M.D. Member of the Royal College of Surgeons, and Surgeon to his Majesty's 7th Regiment of Dragoon Guards.*

IN that excellent Dictionary of Surgery published by Staff-surgeon COOPER, the nature and properties of the buffy coat of the blood are spoken of in very ambiguous terms; so much so, as to leave the subject enveloped in considerable doubt. Its author, in speaking of this substance, under the head of inflammation, says, "In some cases those changes in the blood," (alluding to the production of the buffy coat,) "are deemed a more infallible proof of the existence of inflammation than the state of the pulse itself: they are, however, only a criterion of some unknown operation going on in the system; for the blood taken from a pregnant woman is always found to present the same ap-

* This paper formed a part of the author's annual report to Sir James M'Grigor, written last December.

pearance." The circumstances, also, of the pulse being generally small in peritoneal inflammation, while the buffy coat is found to abound in the blood; and, on the contrary, that, during typhoid diseases, though it may be full and quick, yet true buff cannot be detected; and further, that this substance will be discovered in it at one period of an inflammatory affection, and not at another; and, lastly, no satisfactory explanation of such phenomena being found even in the celebrated works of Hunter, Hewson, Sir Everard Home, Scudamore, &c. have all led me to consider the subject particularly, and to endeavour, in the following pages, to explain the cause of its formation, as well as the uses which it performs in the animal economy under the various circumstances in which it is found to exist.

By the term inflammation it is generally understood that vascular action is increased in the whole or some part of the system, and is accompanied by all or some of the well-known symptoms of that disorder.

It may be divided into three orders, according to the extent of the parts engaged: viz. into *general inflammation*, when the whole system is affected; *mixed*, when a topical affection is accompanied by general symptoms; and *local*, when the disorder is confined to a part, without the participation of the whole system.

These may again be divided into sthenic and asthenic genera, as their nature may be. I would cite pure synocha* as an instance of general inflammation of the sthenic genus, and typhus as the reverse of it; while pneumonia affords an example of sthenic inflammation of the mixed sort, and phlegmon of one purely local.

Some of these affections, particularly of the mixed and local orders, may be still further subdivided into the acute and chronic species. I would quote the pregnant state as an instance of chronic phlegmasia, or mixed inflammation of the chronic species, for reasons hereafter to be given; while most indolent tumors exemplify the local affection of the same nature.

When we shall have examined into the causes, symptoms, and effects of every species of inflammation, from the general, or what is called fever, to the simple whitlow, which may serve to exemplify the purely local affection, we shall find that they are all the effects of efforts which nature makes to relieve the system in general, or some part of it

* I mean the disease described under this head by systematic writers; of which, however, I must confess I never saw an example.

in particular, from the consequences of the application of some offending cause. Thus, inflammatory fever may be excited by cold, or other causes which suppress the accustomed evacuations: this induces local congestions, to relieve which general reaction ensues, and constitutes the disease in question.

This reaction, or fever, then, is the effort which nature makes to relieve itself, while endeavouring to re-establish suppressed evacuations. The specimen of local inflammation, as whitlow, already quoted, will likewise be found to be an effort which nature has made to rid itself from some stimulus, such as a splinter of wood beneath the nail, &c. These efforts will be sthenic in the direct ratio of the natural strength and vigor of the system, or particular parts affected; modified, however, by the destructive powers of the offending causes applied to them. In a healthy subject, when the causes are moderate, sthenic general inflammation, or inflammatory continued fever, is excited; whereas, when the causes which have been applied to the system are highly concentrated and intense, as in some forms of malaria, its powers are so much weakened by their application as to render it incapable of making similar salutary efforts; and death, or asthenic general inflammation, is the consequence, manifested by those genera of disease termed typhoid or remittent fevers.

The system appears likewise to be governed in her salutary efforts by a series of general laws, the whole or some of which are called into action in every species of inflammation, whether local or general; and besides the influence they exert in the increase or decrease of the various secretions, with a view to the re-establishment of the equilibrium in the circulation, they call to their assistance other active processes, some of which act diametrically opposite to each other in their immediate effects. In cases of local inflammation of the sthenic genus, such as phlegmon, and of mixed, as pneumonia, the action of the absorbents is particularly put in requisition, in order either to remove the offending body, or, if necessary, to insulate the affected parts, so as to prevent its admission into the system. While this action, apparently of a destructive nature, is undertaken, the protecting powers of the system become alarmed, and, in order to be in readiness to repair the damage which may be done to it, prepare in the blood the means of reproduction, by inducing the formation of the buffy coat, to enable it to deposit coagulable lymph, wherever it may be found necessary to do so. We observe these two processes, one of increase and the other of decrease, particularly active even in the very common but necessary operations of resolution, ulceration, suppuration, granulation, adhesion, and effusion. Of all the processes which nature has instituted for the purpose of protecting itself against the consequences of inflammation, there is none which exercises a more important office than that which induces the buffy coat in the blood. By it the progress of inflammation is arrested or circumscribed, either by the deposition or effusion of coagulable lymph. We therefore find that, as soon as the system has had sufficient time to sympathize in a case of mixed inflammation, or phlegmasia, for example, what is termed the buffy coat is developed; but it appears that a certain time is necessary for the accomplishment of this object. Thus, blood drawn at the commencement even of pneumonia may not exhibit it. I have often observed this, but more particularly in a very recent case. In it, as long as the system appeared to be prevented from acting with freedom, owing to congestion of blood in the lungs, no buff was observed: I abstracted two pounds in the morning and two pounds more in the evening, with manifest advantage to the general symptoms, but without finding buff. In the course of the next day, having deemed it necessary to have recourse once more to phlebotomy, the blood exhibited the strongest indications of inflammation, as well by the presence of the buffy coat, as by what is termed its cupped appearance. This case would show that, although, when the buffy coat is exhibited by blood drawn in diseases accompanied by other symptoms of inflammation, we are to regard its presence as an absolute proof of the existence of that disorder, either in an acute or chronic form, at the same time its absence, under similar accompanying circumstances, should not lead us to form a contrary conclusion, and hence, perhaps, omit one of the most certain means of counteracting inflammatory action, namely, general or topical bloodletting.

Nature, in the developement of the buffy coat, not only furnishes itself with means of resisting disease, but of assisting most materially in the operation of perpetuating our species. In the state of pregnancy we observe many of the phenomena characteristic of inflammation, so much so as to lead me to consider it, though a natural and healthy process, as a variety of chronic phlegmasia. In it we observe acceleration of the pulse and local distention, attended, generally speaking, with marks of plethora. We also see the absorbent vessels called into action to detach the ovum, and next we find that the system arms itself with coagulable lymph, in what may be called a free state, as is exemplified by the buff exhibited when blood is taken from pregnant women. A very little consideration, however, will lead us to see that this peculiar state is only a very salutary one, and that it is highly necessary to enable the system to supply coagulable lymph, which is indispensable, as well for the adhesion of the ovum to the uterus, on its arrival within its cavity, as to provide for the gradual and daily increase of the fœtus, by the fixation (if I may use the expression) of the free lymph during the developement of its various parts.

We see, then, that the presence of the buffy coat in the blood is only the effect of a salutary effort of the system, either to resist disease, to repair its consequences, or to act a most important part in that most essential process of the animal economy, the perpetuation of our species. It will also have been seen that, although its existence, when accompanied by other marks of inflammatory action, proves the surest and best test of the propriety of exercising sanguineous depletion; yet its absence, when other symptoms of this acute affection manifest themselves, ought not to deter us from having recourse to that most efficient remedy.

Having thus far considered that power of the system exemplified by the developement of the buffy coat in the blood, or what may be termed the material for reparation or reproduction, let us now take a view of a process, which, though it appears to be antagonist to the one just described in its mode of effecting its purposes, is its great ally in the ultimate result which they are both destined to attain: I mean the action of the absorbents in removing superfluous matter, or even in inducing ulceration where nature may wish to insulate a part, or relieve local determination, by allowing effusion to take place from parts labouring under congestion. It would seem that nature, in its preservative efforts, is influenced by a species of instinct arising out of an association of sensations, which calls her resources into action, in a certain order of succession. For example, as soon as tension or swelling takes place, and that the sensation it causes is experienced, the absorbents are called upon to act; soon after which, provision is made in order to be in readiness to reconstruct what they may have carried away, by the developement of the buffy coat in the blood. We have a curious example of this even in some cases of dropsical swellings: *distention** in such instances is generally

* Distention to a certain extent exerts the same stimulating influence on the absorbents as pressure is known to do; but, if it be increased immodesupposed to be caused by an increase of action in the exhalent vessels, to counteract which the absorbents are naturally called upon to use their endeavours to correct the effects of this deviation from healthy action; by association, their ally, the buffy coat, is invited to come to their assistance; and hence may have arisen the idea that dropsy depends on an inflammatory state of the system in general.

Query: May not the buffy coat in this instance be of service in rendering the blood less capable of passing through the capillaries, and thus more likely to retard exhalation?

When a certain action is once induced in a part, producing swelling and determination of blood to it, we often find that, notwithstanding all our endeavours to resolve or discuss such a swelling, as in cases of chronic glandular enlargements in the groin, for example, suppuration will ultimately ensue. This may be explained on a similar principle to that on which John Hunter accounts for the impotence of mercury, which is sometimes experienced by its not preventing the occurrence of secondary symptoms of the venereal disease, if not used early; namely, that when the disposition to induce them is once formed, nature will not be thwarted in her intentions.

I have more than once observed the truth of this axiom rately, instead of exciting, it paralyses, their action. In this way may we account why general bleeding, by lessening the volume of the circulating mass, and thereby relieving over-distention, may prove apparently, and in some cases really, serviceable in certain stages of dropsy. Magendie drew conclusions of the same practical utility from another chain of reasoning. He supposed that the action of the absorbents was diminished by the existence of plethora, and vice versâ. Thus, when he induced artificial plethora by the injection of water into the veins, he could apply poisons to the pleura and stomach almost with impunity; while, on the contrary, when he bled the subject of his experiments, so as to diminish the circulating mass, it very soon fell a victim to the effects of poisonous absorption. Magendie's experiments are only further proofs of the wisdom with which nature has instituted general laws for the preservation of life. It appears also natural that, when the vessels are distended with water, or that plethora exists, she must feel conscious of the impropriety of increasing the action of the absorbents, and vice versâ. On the same principle, we see the lungs increase or decrease their consumption of oxygen according to the nature of our aliment.

Dr. Paris, in his Pharmacologia, vol. i. 6th edition, page 213, says, "Mr. Spalding, the celebrated diver, found that, when he ate animal food and drank spirits, he consumed in a much shorter time the oxygen in his diving bell, and therefore confined himself to water and vegetables when following his profession. Nature, in this case, exercises her discriminating powers, arising out of an association of sensations, and finding that animal food and spirit contained less oxygen than vegetables and water, induces the lungs to endeavour to abstract from the atmosphere more oxygen when the system is supported on the former than on the latter kind of diet. A knowledge of this circumstance is essential, in order to enable us to explain the rationale of our dietetic regulations in disease of the respiratory organs.

exemplified in cases of soldiers who had contracted the venereal disease, and who had remained without treatment for some time, owing to their absence from the regiment. In such cases, mercury, though administered with care, did not, generally speaking, prevent the appearance of secondary symptoms. On the same principle, then, should the first means employed not succeed in arresting the progress of inflammation, and should nature determine on, or form the disposition to, having recourse to ulceration and suppuration, in cases of glandular swellings, it will be found extremely difficult, and very often impossible, to avert her from her purpose.

In consequence of this difficulty, and from having observed the good effects of repeated blisters, when applied to indolently inflamed tumors, which I imagine could only have acted by inducing ulceration and suppuration, and thus artificially causing, or anticipating, what nature intended to perform; I was led to adopt the practice of exciting ulceration of the integuments immediately above such tumors, by the application of the potassa fusa, and in this way I have succeeded in discussing, quickly, tumors which otherwise must have been obstinate and tedious. When an eschar is formed by the potassa, nature, from its perception of the necessity for the absorbents to act in detaching and removing the slough, calls them to her aid, and, when once they are put in action, they appear to take up whatever superfluous matter they find in their neighbourhood, and thus remove the whole of such tumors. By this means, in most instances we avoid the painful, and often slow, process of internal suppuration, and have, in general, only to await the healing of the artificial sore, which proceeds kindly.

Therefore, as we cannot prevent the supervention of ulceration and suppuration, when the disposition to it is once formed in the system, we must see the propriety of making an early eschar on the integuments over such tumors.

The moxa and issues, in disease of the hip joint, of the spine, and white swellings, must owe their just reputation to the effects they induce: on the same principles, I would, therefore, propose this application to all buboes and similar swellings, as soon as the other means usually prescribed in such cases appear to have failed in inducing resolution, by which we shall generally find that internal suppuration will be avoided.

When the absorbents are once roused into action, they do not confine themselves, as I before mentioned, to the mere extent of the eschar, but extend their influence to the neighbouring parts, in the same way as the system acts when it sets about replacing the loss of blood induced by a small bleeding from the arm; not ceasing its efforts exactly when it has replaced the quantity of blood lost, but continuing to prepare more, and to induce even a fuller state of the vessels than existed previous to the evacuation; thus proving the truth of the popular observation that "bleeding fattens."

By rubbing a small extent of surface with the point of a piece of the potassa fusa, an eschar is very soon formed, and the pain attendant on this operation may then be instantly arrested, by pouring from a little elevation a stream of cold water on the part. The cold water not only causes the pain to cease, but seems to accelerate the death of the part exposed to the action of the potassa. Should the water be slightly acidulated, it will tend to hasten these results.

In typhoid and remittent fevers, and all diseases of increased vascular action, of the asthenic genus, we find that, although the absorbents may be called into action in the efforts which nature may make for the preservation of the system, and that ulceration, or even passive hemorrhages, may be the consequence, yet she is unable to develope the characters of the buffy coat in the blood. On the contrary, principles of a directly opposite nature are found to predominate in it; such as an excess of carbon, and of all other elements which are wont to be separated from the circulation by a vigorous and healthy [redacted]rspiration. re

Thus we observe a marked difference to exist in the qualities of the blood in sthenic and asthenic disease. In the latter affections another mode of defence is set up: a succession of paroxysms, more or less distinct, are induced, during which nature strains every nerve to re-establish, by these means, the lost equilibrium of action in the system.

From our observation, therefore, of the different modes by which nature attempts to relieve herself from the effects of the various diseases by which she is liable to be assailed, in the form of increased vascular action, as well as from our knowledge of the very opposite principles developed in the blood, or "pabulum vitæ," during these struggles, we may lay it down as a general principle, that in sthenic affections, while we ought never to lose sight of the other important auxiliaries which therapeutics point out for their treatment, our chief reliance should be placed on bloodletting, local or general, or both, according to the nature of the case.

In the asthenic genus, on the contrary, or what may be termed paroxysmal diseases, bleeding should only be instituted to relieve congestion, and that not without a considerable degree of caution; while our principal efforts should be directed to the re-establishment of the natural secretions of the system, and to support it by the administration of tonics and mild stimulants, so as to assist in the periodical struggles it may make to get rid of the superabundance of asthenic principles with which the blood abounds in such cases, and to enable it, through the instrumentality of the lungs and other important organs to recover the due proportion of oxygen and vigor which are necessary to the state of health.

*Ipswich Barracks; Sept. 1st, 1829.*

