# Erratum

**Published:** 1829-12

**Authors:** 


ERRATUM. At page 407, line 29, for "perspiration" read "respiration."

